# Early emotional caregiving environment and associations with memory performance and hippocampal volume in adolescents with prenatal drug exposure

**DOI:** 10.3389/fnbeh.2023.1238172

**Published:** 2023-11-20

**Authors:** Brooke H. Kohn, Zehua Cui, Margo A. Candelaria, Stacy Buckingham-Howes, Maureen M. Black, Tracy Riggins

**Affiliations:** ^1^Department of Psychology, University of Maryland, College Park, MD, United States; ^2^Institute for Innovation and Implementation, University of Maryland School of Social Work, Baltimore, MD, United States; ^3^Maryland Health Care Commission, Baltimore, MD, United States; ^4^Department of Pediatrics, University of Maryland School of Medicine, Baltimore, MD, United States; ^5^RTI International, Research Triangle Part, Durham, NC, United States

**Keywords:** caregiving, prenatal drug exposure, development, memory, hippocampus, adolescent brain

## Abstract

Early adversities, including prenatal drug exposure (PDE) and a negative postnatal emotional caregiving environment, impact children’s long-term development. The protracted developmental course of memory and its underlying neural systems offer a valuable framework for understanding the longitudinal associations of pre- and postnatal factors on children with PDE. This study longitudinally examines memory and hippocampal development in 69 parent–child dyads to investigate how the early caregiving emotional environment affects children with PDE’s neural and cognitive systems. Measures of physical health, drug exposure, caregiver stress, depression, and distress were collected between 0 and 24 months At age 14 years, adolescents completed multiple measures of episodic memory, and at ages 14 and 18 years, adolescents underwent magnetic resonance imaging (MRI) scans. Latent constructs of episodic memory and the caregiving environment were created using Confirmatory Factor Analysis. Multiple regressions revealed a negative emotional caregiving environment during infancy was associated with poor memory performance and smaller left hippocampal volumes at 14 years. Better memory performance at 14 years predicted larger right hippocampal volume at 18 years. At 18 years, the association between the emotional caregiving environment and hippocampal volume was moderated by sex, such that a negative emotional caregiving environment was associated with larger left hippocampal volumes in males but not females. Findings suggest that the postnatal caregiving environment may modulate the effects of PDE across development, influencing neurocognitive development.

## Introduction

Drug use during pregnancy is a serious and increasing health problem with risks for both the mother and the unborn child (Richardson and Day, 2018; SAMHSA, 2021). Drug use has risen progressively among pregnant people in the United States, with approximately 60% of pregnant mothers reporting prescription drug use and 7.7% reporting illicit substance use during pregnancy (Traccis et al., 2020; SAMHSA, 2021).

Prenatal drug exposure (PDE, specified here as exposure to cocaine and/or heroin, commonly along with the use of other substances) is associated with adverse developmental outcomes across multiple domains (Morey et al., 2009; Buckingham-Howes et al., 2013). However, longitudinal studies of the impact of PDE on cognitive development remain limited, have shown mixed associations as children age, and have inconsistently evaluated the relative impact of the caregiving context (Ackerman et al., 2010; Riggins et al., 2012; Conradt et al., 2019). A recent systematic literature review found 27 studies on the cognitive outcomes of children with PDE beyond age two (Conradt et al., 2019). Across these studies, some reported no cognitive differences between exposed and non-exposed children, whereas others found differences in IQ and memory performance (Conradt et al., 2019). Longitudinal investigations into the role of biological sex on the impact of PDE suggest that the severity of cognitive and behavioral outcomes and neurodevelopmental consequences may be moderated by sex, such that female sex is protective against adverse cognitive and behavioral outcomes (Suffet and Brotman, 1984; Moe and Slinning, 2001; Bennett et al., 2002, 2007; Delaney-Black et al., 2004; Nair et al., 2008; Nygaard et al., 2015; Conradt et al., 2018; Skumlien et al., 2020; Traccis et al., 2020).

It is highly probable that some individual differences in the long-term outcomes of those with PDE are related to variations in the postnatal environment. The small body of literature on long-term outcomes for children with PDE increasingly points toward a complex story in which postnatal environmental interactions may emerge or recede throughout development and may be sex-dependent (Ackerman et al., 2010; Betancourt et al., 2011; Traccis et al., 2020).

As cognitive development interacts with the social environment and develops over time, understanding the impact of PDE on cognitive and neural development necessitates the longitudinal study of these constructs within their social context. Variations in caregiver emotional state and caregiving are of particular interest, as mental health is associated with variations in caregiving and both positive (e.g., sensitive, responsive, and supportive) and negative^1^ (e.g., intrusive, inconsistent, or insensitive) caregiving plays a role in structuring biological, behavioral, and cognitive development (Nelson, 1993; Cleveland and Reese, 2005; Farah et al., 2008; Faure et al., 2017; Botdorf et al., 2019). The current study focuses on the early emotional caregiving environment (ECE), defined as caregiver’s emotional wellbeing and emotions toward parenting. We examine the interactions between early ECE and later cognitive ability (memory) and its underlying neural correlates, which are particularly susceptible to pre- and postnatal stressors due to their protracted development. These constructs may inform our understanding of the interaction between PDE and environmental risk across development (Geng et al., 2018; Riggins et al., 2018; Botdorf et al., 2019; Canada et al., 2022).

### Protracted development of memory and the hippocampus

#### Typical development

Episodic memory is a cornerstone ability that supports the recall of past events and their spatiotemporal context (Canada et al., 2022). Episodic memory is implicated in academic and socioemotional outcomes and is susceptible to impairment across various psychiatric and neurologic disorders (Ghetti and Bunge, 2012). Memory capacity shows a protracted, linear development course, consistently improving between ages 4 and 8 years (Canada et al., 2022) and into adolescence (Ghetti and Bunge, 2012). Memory is primarily supported by the hippocampus, which is also involved in the stress response and stress/emotion regulation (Herman et al., 2005). The hippocampus also shows a protracted developmental course, maturing and changing structurally throughout adolescence (Ghetti and Bunge, 2012; Geng et al., 2018; Riggins et al., 2018; Botdorf et al., 2019) with normative hippocampal development including both increases and decreases in volume (Botdorf et al., 2019).

The protracted developmental course of both episodic memory and hippocampal development means that differences in functionality and capacity may continue to develop until the supporting neural systems fully develop (Geng et al., 2018; Riggins et al., 2018; Botdorf et al., 2019; Canada et al., 2022). Moreover, this implies an increased time window in which memory and its underlying neural networks are susceptible to environmental influences (Gogtay et al., 2006). For example, a study examining PDE’s effects on incidental memory found that although there were only marginal differences between PDE and non-exposed groups’ performance, memory ability improved at a significantly slower rate in the PDE group (Betancourt et al., 2011). Thus, the impact of PDE on underlying systems may vary over time, suggesting that children may “grow into” or “out of” an impairment as they mature and develop. Therefore, studying the longitudinal development of memory and the hippocampus in this population may offer insight into how PDE interacts with the postnatal social environment across development.

#### Individuals with a history of prenatal drug exposure

To our knowledge, eight studies have specifically explored the cognitive domain of episodic memory among children with PDE histories (Guo et al., 1994; Hurt et al., 2009; Betancourt et al., 2011; Riggins et al., 2012; Sundelin Wahlsten and Sarman, 2013; Konijnenberg et al., 2016; Geng et al., 2018; Konijnenberg and Melinder, 2022). Collectively, these studies encompass an extensive age range (3–17 years), utilize eight different assessments of memory, and are characterized by mixed findings throughout development. Compared to controls, studies report differences in memory performance in early (Sundelin Wahlsten and Sarman, 2013; Konijnenberg et al., 2016) and middle childhood (Guo et al., 1994; Konijnenberg and Melinder, 2022), both differences (Riggins et al., 2012) and no differences (Hurt et al., 2009; Betancourt et al., 2011) in memory performance in early to mid-adolescence, and no differences in memory performance in late adolescence (Betancourt et al., 2011; Geng et al., 2018) (see Supplementary material for a detailed overview). Taken together, these findings suggest that the impact of PDE on memory performance may vary across development.

Both structural and functional magnetic resonance imaging (MRI) studies have reported PDE-associated differences in the neural correlates of memory utilizing the same sample as the present study. Geng et al. (2018) explored the impact of PDE on neural function during recall tasks, reporting hemispheric differences in hippocampal activation between PDE and comparison groups during memory encoding but not retrieval at age 17. Regarding hippocampal structure, Riggins et al. (2012) found PDE was associated with larger hippocampal volumes at age 14 and that larger hippocampal volumes were associated with lower memory scores in a sample of adolescents with and without histories of PDE (Riggins et al., 2012). While these studies show neural differences related to PDE, given the developmental course of memory and the hippocampus, further investigation is needed to better understand how the effects of PDE on brain structure change with age.

Overall, mixed findings across studies may be a function of differences in task performance demands or PDE’s impact changing as episodic memory and its underlying neural systems develop and interact with the social environment throughout early life. This suggests a need for research exploring these constructs more comprehensively across different time points in development.

### Caregiving, memory, and the hippocampus

#### Typical development

Caregiving and ECE represent potential sources of variability that may contribute to individual differences in memory capacity (Farah et al., 2008; Valentino et al., 2009; Larkina and Bauer, 2010; Botdorf et al., 2019) and hippocampal volume (Rao et al., 2010; Luby et al., 2013; Blankenship et al., 2019). Disruptions in caregiving behavior associated with maternal mental health are well documented (Field, 1994; Lovejoy et al., 2000; Pettit et al., 2008; Botdorf et al., 2019; Urizar and Muñoz, 2022). Literature suggests that more negative caregiver emotional state puts a child at greater risk for experiencing negative caregiving characterized by greater negative affect, reduced support, and less sensitivity to child needs (Lovejoy et al., 2000; Dougherty et al., 2013).

Extant literature has demonstrated that memory and the hippocampus are sensitive to the influence of caregiving among children without PDE (Moore and Zoellner, 2007; Valentino et al., 2009; Valentino, 2011; Luby et al., 2013; Woody et al., 2015), and childhood may constitute a sensitive period for these influences (Rao et al., 2010; Luby et al., 2016). In studies with non-prenatally drug-exposed children, supportive and engaged caregiving has been associated with better autobiographical and general episodic memory (Valentino et al., 2009; Larkina and Bauer, 2010), whereas extreme negative caregiving and maternal depression have been linked to memory deficits such as overgeneralized autobiographical memory (Moore and Zoellner, 2007; Valentino et al., 2009; Valentino, 2011; Woody et al., 2015).

Relatedly, both positive and negative caregiving practices are thought to differentially impact hippocampal volume by altering the neurodevelopmental process of synaptogenesis (Liu et al., 2000). Early experiences of negative caregiving, maternal depression, and high stress in early childhood predict differences in hippocampal structure and function in later childhood (Blankenship et al., 2019; Luby et al., 2019). Researchers have found smaller hippocampal volumes for children who were physically abused or had greater cumulative stress exposure (Hanson et al., 2015; Humphreys et al., 2019; Botdorf et al., 2022). Another study reports significant associations between lifetime stress severity and left hippocampal volume in children younger than five, and no significant association between stress severity and hippocampal volume after age six (Humphreys et al., 2019). Moreover, stress severity in early childhood remained a significant predictor of left hippocampal volume beyond later stress severity–further suggesting evidence for a sensitive period for the effects of life stress on hippocampal volume (Humphreys et al., 2019).

Although more negative caregiving environments and more a negative caregiver emotional state may represent a significant risk factor for differences in hippocampal development, studies report that high-quality caregiving may be particularly beneficial or protective (Luby et al., 2013, 2016, 2019; Wang et al., 2014, 2019). Overall, better caregiving quality is associated with larger left hippocampi and smaller right hippocampi in infancy (Qiu et al., 2013), greater anterior functional connectivity in early childhood (Wang et al., 2019), faster growth in volume at preschool age (Luby et al., 2016), and larger volumes and functional networks during school age (Luby et al., 2013, 2016, 2019; Wang et al., 2014). Findings are mixed in adolescence and young adulthood, as histories of early life adversity and negative caregiving have been associated with both smaller (Bremner et al., 1997; Stein et al., 1997; Vythilingam et al., 2002; Buss et al., 2007; Rao et al., 2010) and larger (Rao et al., 2010) hippocampi during adolescence (Belsky and de Haan, 2011). The impact of high-quality caregiving has also been indirectly associated with hippocampal structure, with some studies showing caregiver support mediating the effects of poverty and preschool Adverse Life Experiences (ACES) on bilateral hippocampal volume (Luby et al., 2013, 2019). Moreover, sex differences have been reported in hippocampal susceptibility to prenatal stress such that male sex is associated with a greater reduction in hippocampal volume (Buss et al., 2007; Samplin et al., 2013; Teicher et al., 2018) and, relative susceptibility may be modulated by the postnatal environment in a sex-specific manner (Buss et al., 2007). This finding suggests positive caregiving may buffer systemic stressors, biological risk, and hippocampal development (Luby et al., 2013, 2019).

Overall, given the protracted development and sensitivity of memory and the hippocampus to early life stress, investigations into the longitudinal development of memory and the hippocampus may be leveraged to provide insight into the impact of postnatal social environments on cognitive development among children with PDE.

#### Individuals with a history of prenatal drug exposure

The impact of PDE on children’s neurocognitive systems varies with exposure to multiple risk and protective factors throughout development (Ackerman et al., 2010; Rao et al., 2010; Buckingham-Howes et al., 2013). A growing literature supports models of heterogeneity in susceptibility to environmental risk that examine the possibility that teratogenic and maternal risk factors interact to influence child behavioral outcomes (Konijnenberg et al., 2015; Schuetze et al., 2021). Overall, the literature suggests that a history of PDE is associated with potential risk factors that may contribute to a negative caregiving environment and, in turn, have negative developmental consequences (Conradt et al., 2023).

Drug use during pregnancy is associated with significant maternal PTSD symptoms, increased likelihood of violence exposure, and higher incidence of psychopathology (Min et al., 2018; Punamäki et al., 2021). Mothers in treatment have described worsening depression and anxiety after delivery and its detrimental effects on their recovery and self-efficacy in caring for their children (Salo et al., 2009; Martin et al., 2022). Substance use may also impact how parents process and respond to infant cues, making caregiving more difficult (Rutherford et al., 2011, 2013, 2020; Parolin and Simonelli, 2016; Kohl et al., 2017; Daigle et al., 2020; Lowell et al., 2022). In addition, infants with histories of PDE may be particularly challenging to care for, with high-pitched, piercing cries, feeding difficulties, and slow, negative responses to stimuli (Coles and Platzman, 1993; Conradt et al., 2019). Therefore, parents who may already face increased neurobiological challenges to caregiving are charged with providing care for infants who are particularly difficult to support.

For children with PDE, repeatedly impaired responses to distress during infancy may set the stage for decreased self-regulatory capacities (Baker, 2018; Morawska et al., 2019). Moreover, PDE among children is often accompanied by additional family and environmental risk factors such as poverty, maternal psychopathology, problematic parent–child interactions, and formal and informal caregiver changes, often resulting in an unstable caregiving environment (Kettinger et al., 2000; Eiden et al., 2014). These environmental risk factors may accentuate the negative effects of PDE on child development through direct or indirect mechanisms (Ackerman et al., 2010; Konijnenberg et al., 2015). Some studies have found that the caregiving environment may exacerbate or buffer against early biological vulnerability due to PDE (Konijnenberg et al., 2015; Jaekel et al., 2021). Postnatal and family factors may account for approximately half of the differences in opioid-exposed and non-exposed children’s cognitive and motor outcomes (Levine et al., 2021). Moreover, the quality of caregiving and home environment at 18 months may mediate the impact of PDE on language development at age 4.5 years (Kim et al., 2021). Overall, these findings suggest that a more favorable early caregiving environment may attenuate the impact of PDE on developmental outcomes. In contrast, a more negative caregiving environment may exacerbate the overall impact of PDE on child development (Conradt et al., 2018, 2023).

Although these findings support the developmental consequences of a significant interaction between PDE and the caregiving environment, the impact of this relationship on memory and its underlying neural structures is primarily unexplored as children age (Ackerman et al., 2010).

Of the eight studies that have specifically focused on the cognitive domain of episodic memory among those with PDE histories, only two directly explored the impact of the caregiving environment on memory outcomes (Guo et al., 1994; Konijnenberg et al., 2016). Konijnenberg et al. (2016) found a significant main effect of mother–child interaction quality on narrative memory at age 4; however, there was no interaction effect of group status and mother–child interaction, suggesting that caregiver-child interaction quality had a similar influence on narrative memory development, regardless of PDE exposure. Similarly, Guo et al. (1994) found that children with PDE histories showed similar reduced memory performance and ERP amplitude to children without PDE histories but currently living with a caregiver using opiates. This suggests that the postnatal environment may have similar adverse effects, regardless of exposure status.

Other studies covaried caregiver environment variables. Two studies found no main memory effect of parental nurturance and environmental stimulation, measured at ages 4 and 8, or maternal depression and foster care placement, measured at child age 11 (Hurt et al., 2009; Betancourt et al., 2011). One study found a significant main effect of maternal depression, measured at child age 6, on immediate recall and left hippocampal volume (Riggins et al., 2012). In this study, more significant depression was related to larger left hippocampal volumes and better immediate recall at age 14 (Riggins et al., 2012).

Collectively, the impact of PDE on child cognitive and neural development is complex, open to environmental influence, and potentially age and sex-dependent. Prior studies highlight the importance of the early caregiving environment for memory and hippocampal development, suggesting the potential of the caregiving environment to either compound or remediate the adverse impacts of PDE on developmental outcomes as children age.

The current study aims to leverage the study of memory and hippocampal development to explore whether the caregiving emotional environment may modulate the impact of PDE on neural and cognitive systems. In line with previous literature, we hypothesize that among children with PDE, a negative caregiving environment during infancy will predict worse episodic memory performance at 14 years (Guo et al., 1994; Konijnenberg et al., 2016) and smaller hippocampal volumes (Bremner et al., 1997; Stein et al., 1997; Vythilingam et al., 2002; Buss et al., 2007; Rao et al., 2010; Belsky and de Haan, 2011; Blankenship et al., 2019) at both 14 and 18 years. We further hypothesize that poor episodic memory at age 14 will be associated with variations in hippocampal volumes at ages 14 and 18 (Riggins et al., 2012). Moreover, we propose that these associations will be moderated by biological sex, such that males will have worse memory performance and larger hippocampal volumes than females (Bennett et al., 2002; Buss et al., 2007; Nair et al., 2008; Samplin et al., 2013; Traccis et al., 2020).

## Methods

### Participants

#### Phase 1 (Prenatal-24 months Postpartum)

Participants were drawn from a randomized, controlled trial of a home-based intervention for substance-using women and their infants (Nair et al., 2008). Two hundred sixty five mothers (Age *M =* 26.89*, SD* = 5.21) were recruited from an urban University Hospital and enrolled at delivery in the early 1990s (Schuler et al., 2000). Recruitment procedures have been reported previously (Schuler et al., 2002).

Parent–child dyads were randomized to intervention and control groups. Eligibility included gestational age > 32 weeks, birth weight > 1,750 g, no neonatal intensive care unit admission, and positive (cocaine/heroin) maternal/infant urine toxicology or maternal self-report of cocaine/heroin use during pregnancy. Intervention families received developmentally oriented home visits for one year. See Table 1 for complete maternal demographics.

**Table 1:** Demographics of mothers retained for analyses.

Demographics of mothers retained for analyses	
Mothers (*N* = 69)	Mean (SD)
Age at baseline (years)	27.53 (4.94)
Age at birth of first child (years)	19.03 (4.97)
Race (%)	92.75% Black
Number of pregnancies	3.74 (2.55), Range = 1–17
Maternal education in years	11.16 (1.56)
Mothers who completed any post-secondary education (%)	7.25%
Mothers who were never married (%)	72.46%
Families receiving public assistance (%)	60.87%

A pediatrician reviewed neonatal medical records for head circumference, length, birth weight, gestational age, length of hospital stays, birth asphyxia, respiratory distress, sepsis, and neonatal abstinence syndrome (NAS). The mean gestational age was 38.45 (*SD* = 2.52) weeks, the mean length of hospital stay was 5.07 days (*SD* = 4.37), and the average birth weight was 2750.19 g (SD = 468.02). The total number of total neonatal problems ranged from 0 (26.01%), to 1 (18.84%), to ≥2 (26.01%), (*M* = 1.86, SD = 1.73), and 26 participants (37.68%) received a diagnosis of NAS. See Table 2 for birth outcomes.

**Table 2:** Child demographic and descriptive data.

Child demographics			
Time point:	Phase 2: Early adolescence (*N* = 69) *(M(SD))*	Phase 2: Early adolescent imaging (*n* = 27) *(M(SD))*	Phase 3: Late adolescence (*n* = 17) *(M(SD))*
Age *(Years)*	14.24 (1.14)	14.51 (1.18)	18.1(1)
Biological sex (%)	52.17% Female	54.85% Female	58.82% Female
Race (%)	98.55% Black	100% Black	100% Black
*Birth outcomes*			
Gestational age (*Weeks*)	38.45 (2.52)	38.15 (2.51)	38.12 (2.42)
Birth Weight (g)	2750.19 (468.02)	2794.46 (487.81)	2616.94 (510.35)
Length of hospital stay post-delivery (days)	5.07 (4.37)	4.77 (4.25)	5.06 (4.88)
Neonatal abstinence diagnosis (%)	37.68%	23.08%	35.29%
*Substance exposure*			
Exposed to heroin (%)	28.99%	44.44%	47.06%
Exposed to cocaine (%)	14.49%	7.40%	5.88%
Exposed to both cocaine and heroin (%)	52.52%	44.44%	47.06%
Heavy prenatal exposure vs. light (%)	84%	81.48%	82.35%
Exposed to alcohol (%)	53.62%	55.56%	76.47%
Heavy alcohol exposure vs. light (%)	13.51%	2%	15.38%
Exposed to tobacco (%)	81.16%	77.78%	94.12%
Heavy tobacco exposure vs. light (%)	94.64%	95.24%	93.75%
Overall exposure to 3 or more substances (%)*	86.96%	74.35%	76.47%
Received intervention (%)	37.68%	55.56%	58.82%
*Caregiver continuity*			
Number of caregiver changes before age 7	0.94 (1.7)	0.85(1.05)	0.71(1.1)
≥2 caregiver changes (%)	23.19%	22.22%	23.53%

*Reflects prenatal exposure to three or more substances, including opioid and stimulant exposure.

#### Phase 2: early adolescence

Participants were recontacted during adolescence to participate in a follow-up study to assess longitudinal outcomes. Of participants retained through adolescence, 69 provided usable data (Age *M =* 14.24, *SD =* 1.14, 52.17% female). Only participants who provided data at 14 years were retained for analyses.

Of the retained children, 14.49% were exposed to cocaine, 28.99% were exposed to heroin, and 52.52% were exposed to cocaine and heroin. In most cases, exposure to cocaine and/or heroin (84%) was “heavy” as defined by a positive toxicology screen at birth and/or maternal self-reported use of 2 times or more per week during the last six months of pregnancy (i.e., 48–180 days). Consistent with previous studies (Ackerman et al., 2010), other drug use was common (i.e., cigarettes, alcohol); 87% were exposed to 3 or more substances including cocaine and heroin. See Table 2 for child exposures.

Participants in the final sample were compared to participants who were lost to follow-up on the following seven key variables: birth weight, maternal education, maternal age at first pregnancy, maternal age at the birth of the target child, neonatal abstinence scores, child gender, and receipt of public assistance. There were no significant differences between those lost and those retained. See Table 2 for child demographics.

A subset of 27 adolescents were eligible and agreed to participate in the neuroimaging sub-study (Age *M =* 14.51 years, *SD =* 1.18, 54.85% female). The demographic characteristics of participants in the neuroimaging subset were similar to those of the larger sample (Table 2).

#### Phase 3: late adolescence

Participants were recontacted to participate in a follow-up of the neuroimaging study. Seventeen participants were eligible and provided usable data (Age *M =* 18.1 years, *SD =* 1, 58.82% female). 64.71% of participants provided data at both 14- and 18-year scans. The demographic characteristics of the participants were similar to those of the larger sample and the phase 2 imaging participants (Table 2).

## Procedures

PDE was assessed at delivery through a positive maternal toxicology screen, positive infant toxicology screen, maternal self-report, and/or notation in the mother’s medical chart (Black et al., 1993, p. 199; Schuler et al., 2000). All caregivers and their children completed a systematic protocol in the lab. The Institutional Review Boards at the University of Maryland Baltimore and the National Institute on Drug Abuse Intramural Research Program approved the study. Informed consent was obtained from all caregivers, and assent/consent was obtained at adolescence as appropriate (assent for younger than 18 years, consent for 18 years or older).

### Measures

**Figure 1:**
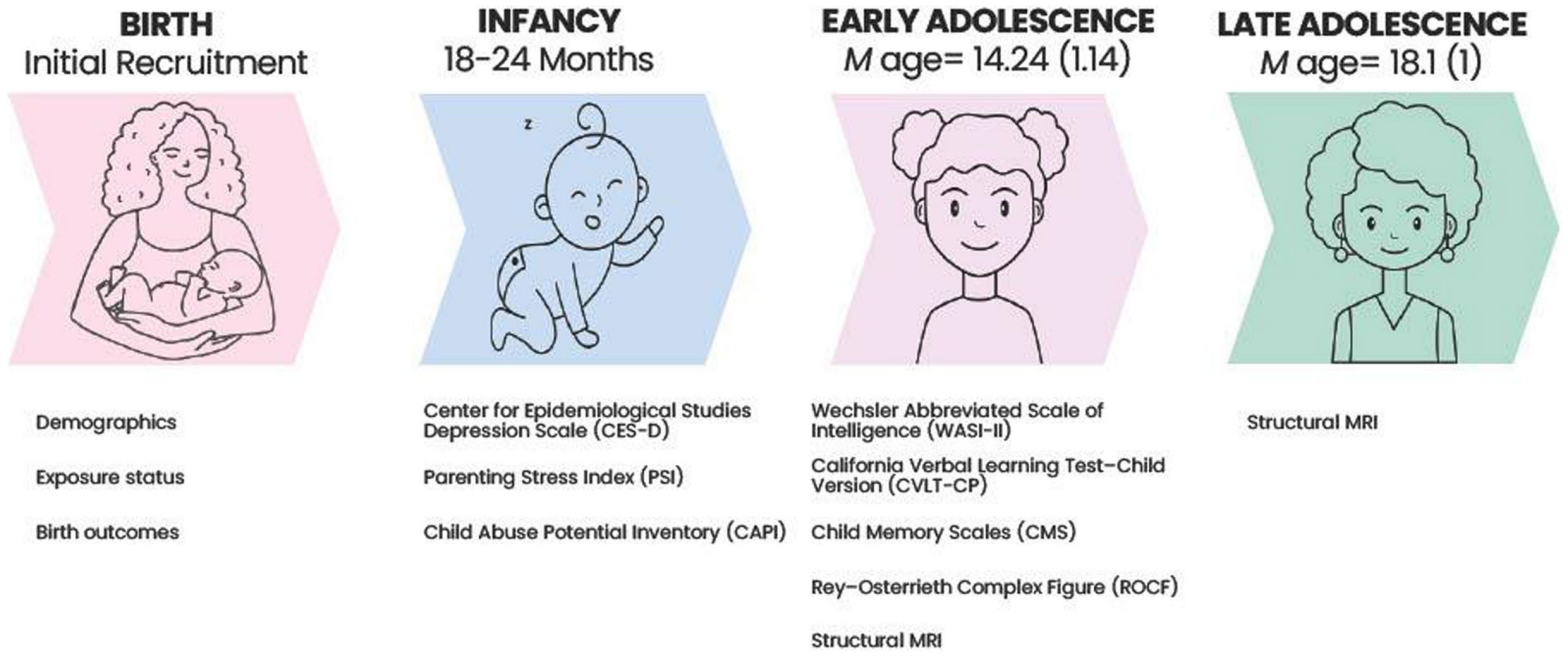
Timeline of study procedures.

See Figure 1 for a timeline of study measures.

#### Phase 1 (Prenatal-24 months postpartum)

##### Caregiver depressive symptoms

The Center for Epidemiological Studies Depression Scale (CES-D), a 20-item self-report depressive symptom scale, was administered at 24 months (Radloff, 1977). Participants rate how often over the past week they have experienced symptoms on a 4-point scale. The CES-D includes items such as “I was bothered by things that usually do not bother me.” The CES-D has demonstrated high internal consistency, reliability, and sensitivity to differences between caregivers and non-caregivers (Pinquart and Sörensen, 2003). Scores for total depressive symptoms ranged from 0 to 39. Cronbach’s alpha for this study was 0.89. Higher scores indicate higher severity of symptoms. Scores >15 are in the clinical range (Radloff, 1977); 27.53% (*n =* 19) of caregivers met the clinical criteria for depression.

##### Caregiver stress

The Parenting Stress Index (PSI), a 101-item scale, was administered to caregivers at 18 months (Abidin, 1995). The PSI is scored on a 5-point Likert scale to tap into the domains of parent characteristics, child characteristics, and situational life stress. The scale is considered the gold standard measure for parental stress. Numerous studies provide evidence of its high internal consistency, good test–retest reliability, and validity in a wide range of at-risk youth populations (Ríos et al., 2022). The total mean parent score was used to indicate overall stress, with higher scores indicating more significant stress (Abidin, 1995). Scores >148 are in the clinical range (Abidin, 1995); 13.04% (*n =* 9) of caregivers met clinical criteria for parental stress.

##### Caregiver distress

The Child Abuse Potential Inventory (CAPI), a 160-item assessment of caregiver inclination toward abuse and neglect, was administered to caregivers at 24 months (Milner, 2004). It has shown good internal consistency and reliability across sample groups and cultures (Walker and Davies, 2010). The CAPI asks participants whether they agree or disagree with a statement to estimate the risk of a parent physically abusing a child. Higher scores indicate a greater likelihood of abuse. The distress factor score was used in analyses. Scores above 152 are in the clinical range (Milner, 2004); 21.74% (*n* = 15) of caregivers met clinical criteria.

##### Caregiver changes

Respondents reported caregiver changes at each evaluation, defined as residing with a non-maternal caregiver for ≥1 month. Caregiver changes by age seven years were summed (*M* = 0.94, SD = 1.7); 44.92% had no caregiver changes, 26.01% had one change, and 23.19% had ≥2 changes.

#### Phase 2: early adolescence

Wechsler Abbreviated Scale of Intelligence (WASI-II) is an abbreviated verbal and nonverbal intelligence measure designed for individuals 6–90 years old. The Full-Scale IQ (FSIQ-2), composed of the matrix reasoning and vocabulary sections, was used in the current study. The FSIQ-2 yields standardized scores. It has been found to have a split-half reliability coefficient of 0.89 and a test–retest reliability of 0.90 (McCrimmon and Smith, 2013).

California Verbal Learning Test–Child Version (CVLT-C; Delis et al., 1988) measures strategies and processes involved in learning and recalling verbal material. Participants are asked to remember a shopping list of 15 items (List A). For the first five trials, the same list was read to participants, and they were asked to recall words from the list after each presentation. A second interference list (List B) was then presented, and participants were asked to recall as many words from this list as possible. When the List B trial was completed, participants were again asked to recall words from List A without an additional presentation of List A. The test has shown high internal consistency and reliability (Mottram and Donders, 2005). Only scores from the free recall, short-delay portion were used in analyses to represent episodic memory.

Child Memory Scales (CMS, Cohen, 1997) measures learning and memory across various dimensions. Participants were read two short stories and asked to recall them immediately and after a 15-min delay. Only scores from the delayed verbatim recall were used in analyses to represent episodic memory. This assessment resulted in measures of immediate and delayed recall of verbatim and thematic information and delayed recognition. CMS has shown high test–retest and split-half reliability and moderate to high concurrent validity (Cohen, 2011).

Rey–Osterrieth Complex Figure (ROCF; Rey and Osterrieth, 1941) has been used to evaluate non-verbal memory and viso-constructional ability for decades. Participants copy complex geometric shapes and reproduce them from memory in delayed recall trials. The Taylor 1959 scoring method was used to evaluate each unit’s accuracy and the figure’s relative position, reflecting the degree of similarity between the original image and the reconstruction (Frisk et al., 2005). Each element of the ROCF was scored between 0 and 2 points. Total accuracy scores range from 0 to 36. Delayed recall scores were used for analyses. The ROFC has shown good clinical practicality, high inter-rater reliability, and good concurrent and content validity (Davies et al., 2011; Zhang et al., 2021).

#### Phases 2 and 3: ealy and late adolescence

##### Hippocampal volume

A 3-T Siemens Allegra scanner was used to acquire a whole-brain oblique axial T1-weighted structural image (MPRAGE) for anatomical evaluation (1-mm^3^ isotropic voxels: TR = 2.5 s; TE = 4.38 ms; FA = 80°). Cortical reconstruction and volumetric segmentation were performed using AFNI (Analysis of Functional Neuro-Imaging; Cox, 1996) and the Freesurfer image analysis suite. The technical details of the Freesurfer pipeline are described in prior publications (Dale and Sereno, 1993; Dale et al., 1999; Fischl et al., 1999, 2001, 2002, 2004; Han et al., 2006, p. 200). Freesurfer morphometric procedures have shown good test–retest reliability across scanner manufacturers and field strengths (Han et al., 2006). Freesurfer has been validated against manual measurements and has shown a reliable ability to detect differences in hippocampal volume (Morey et al., 2009).

## Analytic approach

### Emotional caregiver environment

**Figure 2:**
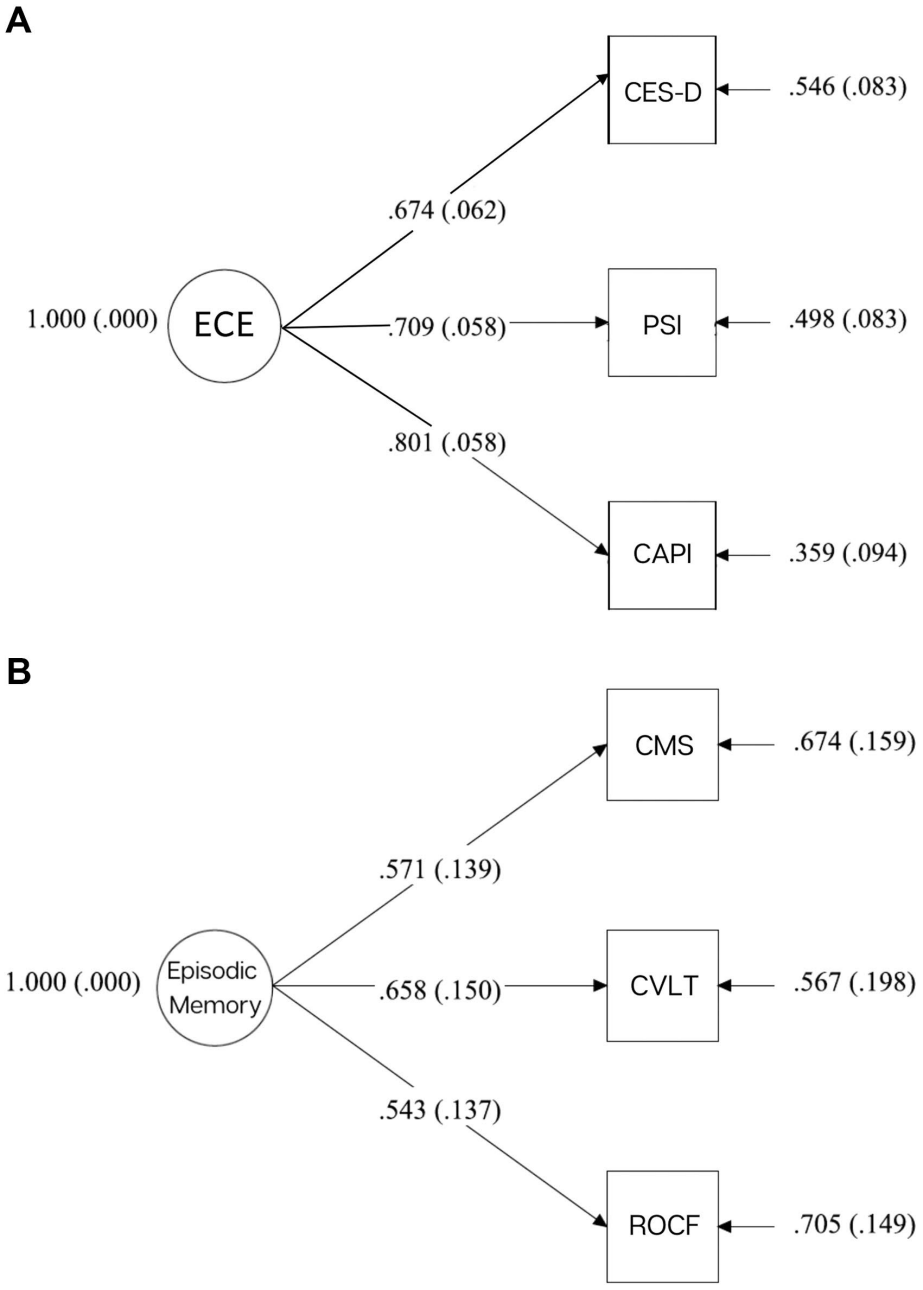
**(A)** Standard factor loadings for ECE. **(B)** Standard factor loadings for episodic memory.

Confirmatory factor analysis (CFA) with Mplus 7.11 was used to construct a latent variable of emotional caregiver environment (ECE), composed of three dimensions from measures collected in Phase 1: maternal depression (CES-D, *M =* 14.41, *SD* = 10.74), maternal stress (PSI: Parent Score, *M =* 131.02, *SD* = 19.43), and caregiver distress (CAPI: Distress Score, CAPI; *M =* 83.06, *SD* = 71.04). Scores were standardized and utilized in a one-factor, just-identified model. Standardized factor loadings range from 0.67–0.80 (caregiver depression: 0.67, *p* < 0.001, caregiver stress: 0.71, *p* < 0.001, caregiver distress: 0.080, *p* < 0.001) (See Figure 2A). High scores represent a negative caregiver emotional state.

### Episodic memory

CFA was used to construct a latent variable of adolescent memory from three different delayed free-recall tasks from three separate Phase 2 measures: the CVLT-C (*M =* 10.38, *SD* = 2.38), the CMS (*M =* 26.18, *SD* = 14.04), and the ROCF task (*M =* 11.64, *SD* = 6.15). Scores were standardized and utilized in a one-factor, just-identified model. Standardized factor loadings range from 0.54 to 0.66 (CVLT-C: 0.66, *p* < 0.001, CMS: 0.57, *p* < 0.001, ROCF: 0.54, *p* < 0.001) (see Figure 2B). High scores indicate better memory performance.

### Hippocampal volume adjustment

Hippocampal volumes were adjusted for age, sex, and intracranial volume (ICV) following methods detailed by Keresztes et al. (2017). In analyses exploring potential moderation by sex, hippocampal volumes were only adjusted for age and ICV so as not to remove for sex-dependent differences.

### Analytic plan

**Figure 3:**
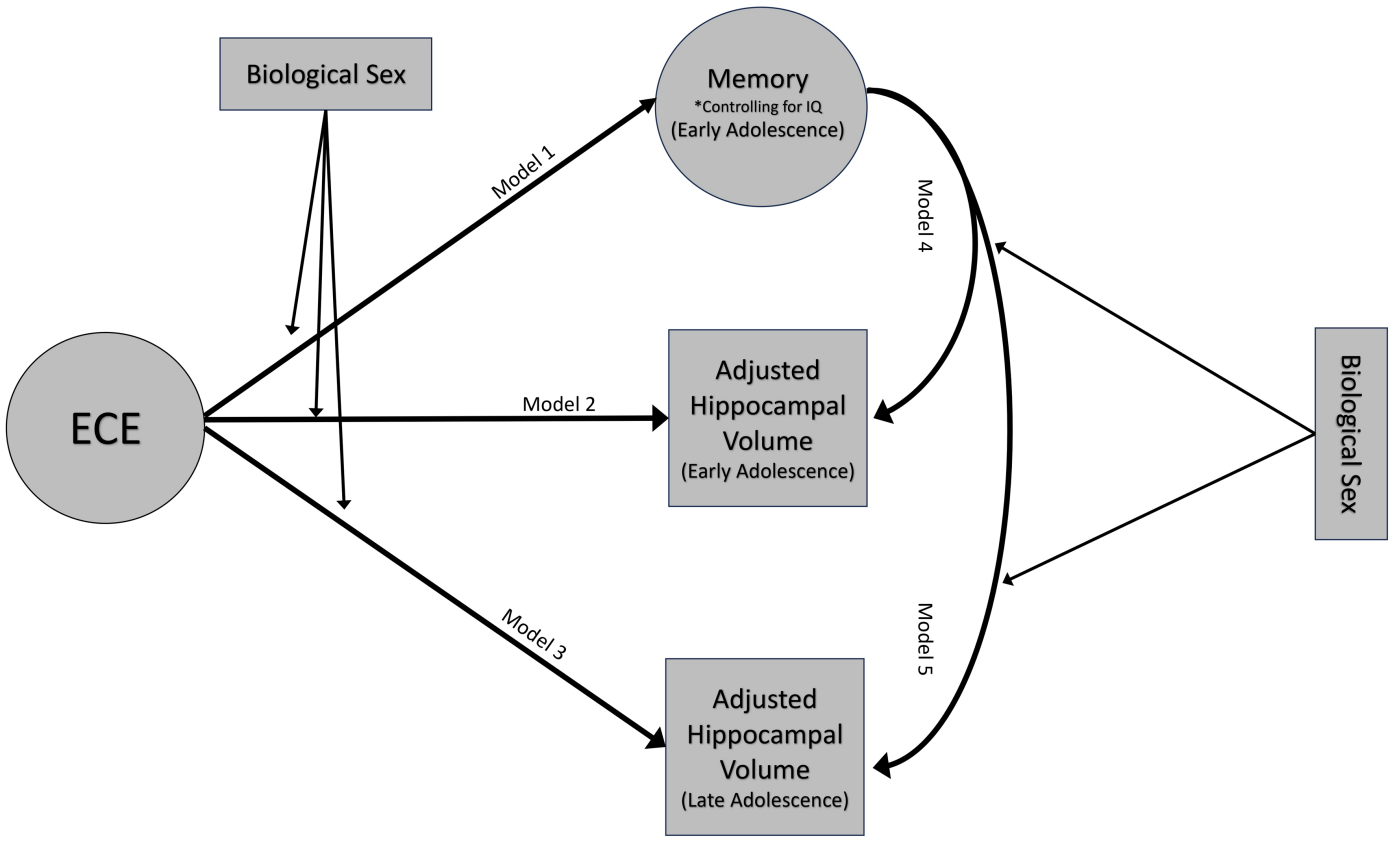
Conceptual model. Emotional caregiving environment will predict episodic memory performance at 14 years (model 1) and hippocampal volumes at both 14 (model 2) and 18 years (model 3). Episodic memory at age 14 will be associated with variations in hippocampal volumes at ages 14 (model 4) and 18 (model 5). These associations will be moderated by biological sex. Moderation analyses only adjusted hippocampal volumes for age and ICV.

Factor scores for latent variables were extracted for analyses. Multiple linear regressions were used to test the associations between the three constructs (Figure 3). Given the relations between the constructs, analyses utilizing episodic memory controlled for FSIQ-2 to obtain a more precise measurement of memory.

After testing the initial relationships, follow up analyses were performed to assess biological sex as a potential moderator.

## Results

**Figure 4:**
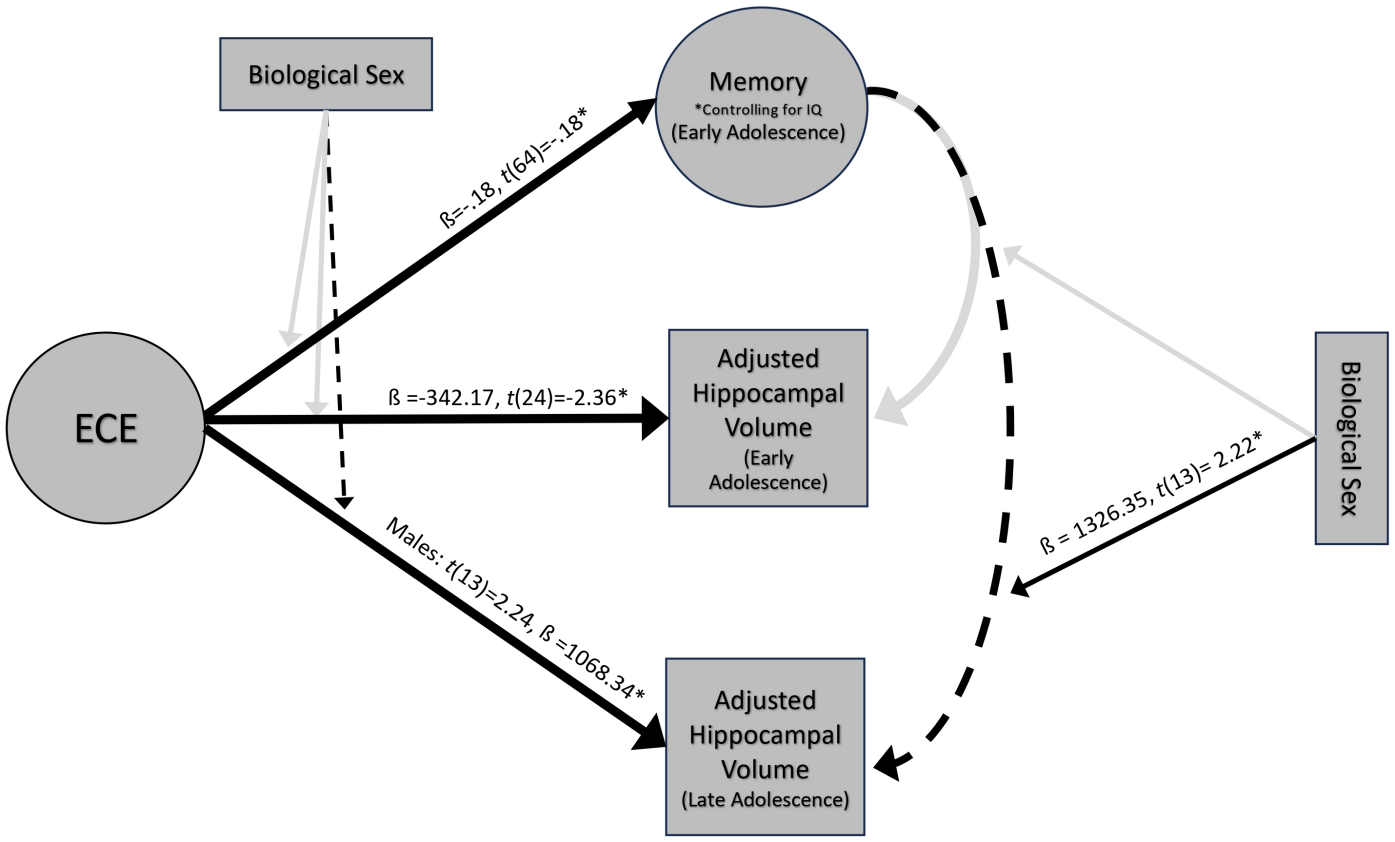
Results of multiple linear regressions at 14 and 18 years. *ps < 0.05, dashed lines indicate marginal significance ps < 0.10.

Results of analyses outlined in the analytic plan are reflected in Figure 4.

### ECE and episodic memory (Model 1)

**Figure 5:**
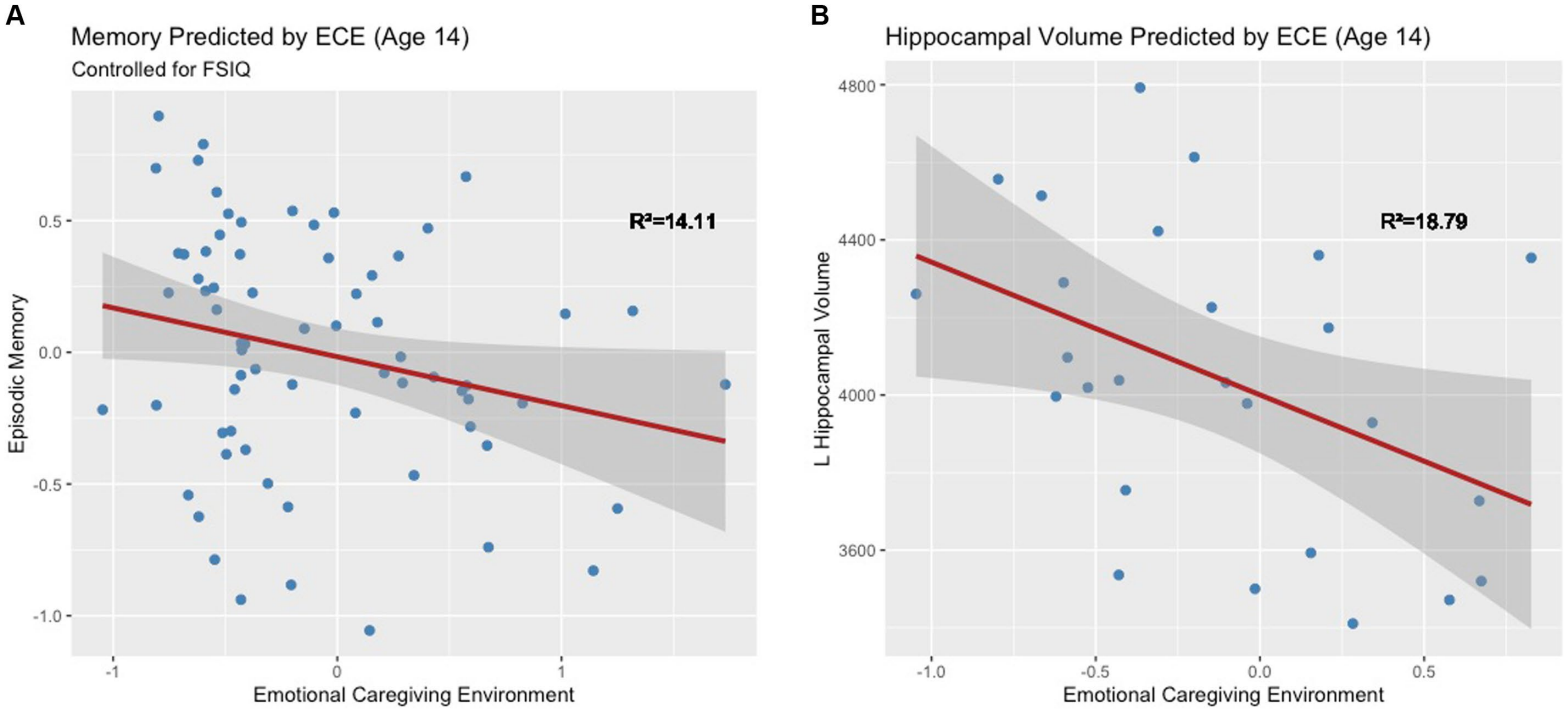
**(A)** Early emotional caregiving environment predicts episodic memory at age 14. **(B)** Early emotional caregiving environment predicts left hippocampal volume at age 14.

The full model explained significant variance in episodic memory scores, R^2^ = 14.11%, *F*(2, 64) = 5.26, *p* = 0.01. Negative ECE was associated with low memory scores when controlling for IQ, ß = −0.18, *t*(64) = −0.18, *p* = 0.04 (Figure 5A). This significant association held after controlling for the number of caregiver changes ß = −0.18, *t*(59) = −0.18, *p* = 0.04. Moderation analyses revealed no significant interaction between ECE and biological sex in predicting episodic memory, *F*(1, 62) = −0.16, *p* = 0.35. Based on a Bayes Factor Analysis, the null hypothesis is 3.64 times more likely than the alternative; thus, we conclude that biological sex does not significantly moderate the association between ECE and episodic memory.

### ECE and early adolescent hippocampal volume (Model 2)

ECE explained significant variance in adjusted left hippocampal volume at age 14, *R*^2^ = 18.79%, *F*(1, 24) = 5.55, *p* = 0.03. Negative ECE was associated with smaller adjusted left hippocampal volume, ß = −342.17, *t*(24) = −2.36, *p* = 0.03 (Figure 5B). This significant association held after controlling for the number of caregiver changes ß = -343.55, *t*(23) = −2.3, *p* = 0.03. ECE did not explain significant variance in right hippocampal volume (*R*^2^ = 6.87%, *F*(1, 24) = 1.77, *p* = 0.20). Moderation analyses revealed that there was not a significant interaction between ECE and biological sex in predicting adjusted left hippocampal volume, *F*(1, 23) = 0.37, *p* = 0.55. Based on a Bayes Factor Analysis, the null hypothesis is 1.92 times more likely than the alternative; thus, we conclude that biological sex does not significantly moderate the association between ECE and left hippocampal volume.

### ECE and late adolescent hippocampal volume (Model 3)

**Figure 6:**
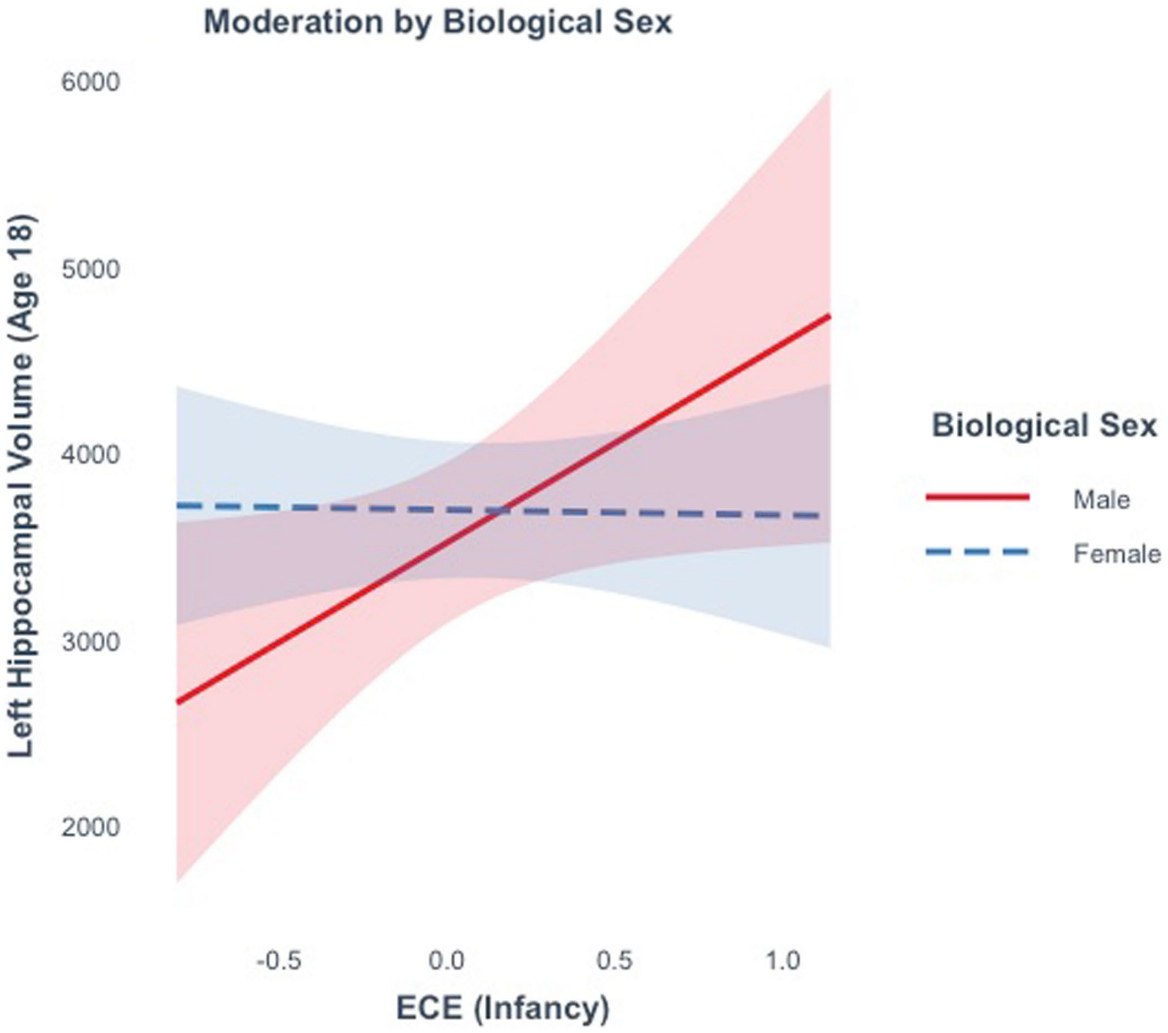
The effect of the early emotional caregiving environment on hippocampal volume at age 18 is moderated by biological sex.

ECE did not explain significant variance in right (*R*^2^ = 0.59%, *F*(1, 15) = 0.09, *p* = 0.77), or left hippocampal volume (*R*^2^ = 6.28%, *F*(1, 15) = 1.00, *p* = 0.33) at age 18. Moderation analyses revealed that there was not a significant interaction between ECE and biological sex in predicting bilateral (ß = −357.7, *t*(13) = −0.40, *p* = 0.69) or right hippocampal volume (ß =755.6, *t*(13) = 1.3, *p* = 0.22). There was a marginally significant interaction between ECE and left hippocampal volume (ß = −1096.7, *t*(13) = −2.0, *p* = 0.06). A negative ECE was associated with larger left hippocampal volumes in males (ß =1068.34, *p* = 0.04), but not in females (ß = -28.35, *p* = 0.92) (Figure 6). In analyses controlling for the interaction, there was a significant main effect of ECE on left hippocampal volume (ß = 1068.3, *t*(13) = 2.24, *p* = 0.04), such that a negative ECE was associated with larger left hippocampal volume.

### Episodic memory and early adolescent hippocampal volume (Model 4)

The full model did not explain a significant amount of variance in right (*R*^2^ = 2.36%, *F*(2, 23) = 0.28, *p* = 0.76) or left hippocampal volume (*R*^2^ = 3.88%, *F*(2, 23) = 0.46, *p* = 0.63) at age 14. Episodic memory (*t*(23) = 0.89, *p* = 0.38) did not significantly predict adjusted left hippocampal volume in early adolescence. Moderation analyses revealed that there was not a significant interaction between memory and biological sex in predicting left hippocampal volume, (*F*(1, 23) = 0.32, *p* = 0.55). Based on a Bayes Factor Analysis, the null hypothesis is 7.05 times more likely than the alternative; thus, we conclude that biological sex does not significantly moderate the association between episodic memory and left hippocampal volume.

### Episodic memory and late adolescent hippocampal volume (Model 5)

**Figure 7:**
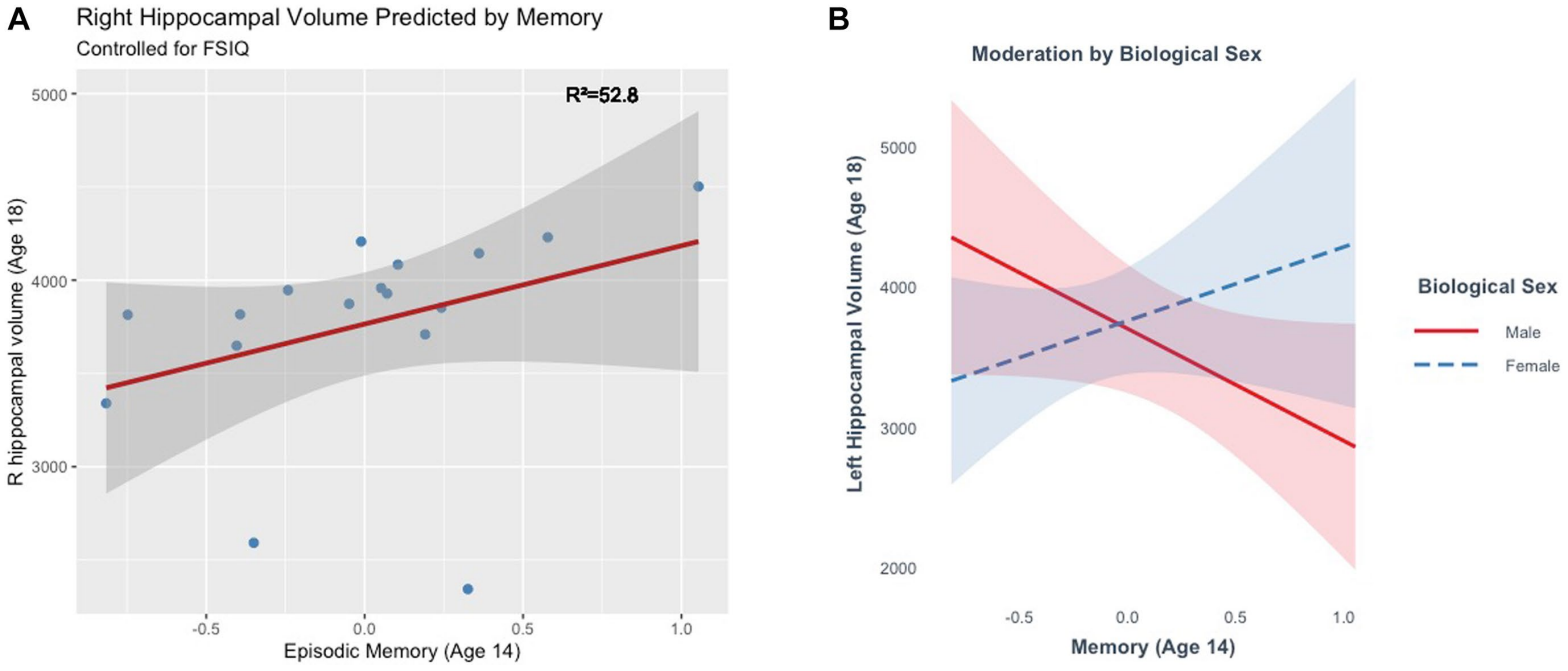
**(A)** Episodic memory at age 14 marginally predicts right hippocampal volume at age 18. **(B)** The effect of episodic memory at age 14 on hippocampal volume at age 18 is moderated by biological sex..

The full model explained a significant amount of variance in right hippocampal volume (*R*^2^ = 52.8%, *F*(2, 13) = 7.27, *p* = 0.01) at age 18. The main effect of memory in this model was marginal, (ß = 431.71, *t*(13) = 1.93, *p* = 0.07), such that better memory scores were marginally associated with larger right hippocampal volumes (Figure 7). The model did not explain a significant amount of variance in bilateral (*R*^2^ = 32.02%, *F*(2, 13) = 3.06, *p* = 0.08) or left hippocampal volume (*R*^2^ = 5.78%, *F*(2, 13) = 0.40, *p* = 0.68). Moderation analyses revealed no significant interaction between memory and biological sex in predicting bilateral (ß =767.8, *t*(13) = 0.80, *p* = 0.44) or right hippocampal volume (ß = −400.9, *t*(13) = −0.62, *p* = 0.55). There was a significant interaction between memory and biological sex in predicting left hippocampal volume (ß = 1326.35, *t*(13) = 2.22, *p* = 0.04). Better memory was marginally associated with smaller hippocampal volumes in males (ß = 800.12, *p* = 0.07), but not in females (ß = 526.23, *p* = 0.25) at age 18 (Figure 7).

### Additional analyses

Pearson’s product moment correlations examining the relationship between neonatal abstinence syndrome (NAS) and outcome variables were nonsignificant. At 14 years, NAS was not significantly correlated with memory *r*(67) = −0.09, *p* = 0.47, ECE *r*(67) = 0.14, *p* = 0.24, bilateral hippocampal volume *r*(24) = 0.129, *p* = 0.53, left hippocampal volume *r*(24) = 0.12, *p* = 0.56, or right hippocampal volume *r*(24) = 0.13, *p* = 0.525. Similarly, at 18 years, NAS was not significantly correlated with bilateral hippocampal volume *r*(15) = 0.27, *p* = 0.3, left hippocampal volume *r*(15) = 0.21, *p* = 0.42, or right hippocampal volume *r*(15) = 0.19, *p* = 0.46.

Pearson’s product moment correlations examining the relationship between intervention group status and outcome variables were nonsignificant. Intervention status was not significantly correlated with ECE, *r*(67) = 0.04, *p* = 0.72. At 14 years, intervention status was not significantly correlated with memory, *r*(67) = −0.09, *p* = 0.45, right hippocampal volume, *r*(24) = −0.013, *p* = 0.52, or left hippocampal volume *r*(24) = −0.19, *p* = 0.36. At 18 years, intervention status was not significantly correlated with right hippocampal volume *r*(15) = −0.19, *p* = 0.46, or left hippocampal volume, *r*(15) = 0.17, *p* = 0.50.

## Discussion

This study explored whether the emotional caregiving environment during infancy may modulate the impact of PDE on neural and cognitive systems at 14 and 18 years. Results show significant associations between early emotional caregiving environment, memory, and hippocampal volume among participants with a history of PDE. In line with the hypotheses and previous literature (Guo et al., 1994; Konijnenberg et al., 2016), at 14 years, a negative emotional caregiving environment during infancy was associated with poor memory capacity, even after controlling for IQ, and with smaller hippocampal volumes in the left hemisphere. Better memory performance at 14 years predicted larger right hippocampal volume at 18 years. At 18 years, the association between the early emotional caregiving environment and hippocampal volume was moderated by sex, such that a negative emotional caregiving environment was associated with larger left hippocampal volumes in males but not females. Taken together, these findings suggest that PDE and the postnatal caregiving environment work together across development to influence neurocognitive systems. Such work is critical as it sheds light on modifiable factors that can buffer the effects of PDE across development.

Several findings are consistent with previous literature. First, in line with Guo et al. (1994) and Konijnenberg et al. (2016), negative ECE during infancy was associated with poorer memory capacity at 14 years, even after controlling for IQ. Second, findings are consistent with previous studies indicating that early ECE is related to smaller hippocampal volumes at 14 years (Bremner et al., 1997; Stein et al., 1997; Vythilingam et al., 2002; Buss et al., 2007; Rao et al., 2010; Belsky and de Haan, 2011; Blankenship et al., 2019). Finally, in line with emerging findings suggesting sex differences in relation to ECE and hippocampal volume, we found that biological sex moderated the association between ECE and hippocampal volume, as well as memory performance and hippocampal volume at age 18 (Moe and Slinning, 2001; Bennett et al., 2002, 2007; Delaney-Black et al., 2004; Buss et al., 2007; Nair et al., 2008; Samplin et al., 2013).

In contrast, some aspects of our findings are contrary to our hypotheses and previous literature. At age 18, our findings did not support a significant association between early emotional caregiving environment and hippocampal volume. However, follow-up moderation analyses found a marginally significant interaction between ECE and left hippocampal volume such that, after controlling for the interaction between ECE and left hippocampal, the main effect of ECE was significant, and a negative ECE was associated with larger left hippocampal volume. The direction of this effect was in the opposite direction than predicted for age 18. This finding contributes to the mixed literature related to the direction of the effect, in line with findings from Rao et al. (2010) but contrary to others who have suggested greater that early-life adversity is associated with smaller hippocampal volumes at age 18 (Bremner et al., 1997; Stein et al., 1997; Vythilingam et al., 2002; Buss et al., 2007; Rao et al., 2010; Belsky and de Haan, 2011; Blankenship et al., 2019). The mixed literature may result from a myriad of factors (see Belsky and de Haan, 2011 for a review). Notably, the studies that have shown an association are not homogenous in their age range, covariates, or conception of early-life stress (e.g., parental psychopathology, abuse, or neglect), which may contribute to some of the variability in findings. Moreover, it is possible that the children who remained in the present study through the 18-year timepoint had the most involved caregivers. This possibility may introduce further variability, as differential effects of optimal positive caregiving versus overprotection have been found on adult hippocampal volume (Wang et al., 2017).

Overall, the finding that negative ECE was associated with smaller left hippocampal volume at age 14 and larger left hippocampal volume in males at age 18 only further supports the theory that the hippocampus remains susceptible to environmental influence throughout adolescent development and the effects of the early social environment may be age-related (Gogtay et al., 2006). The variation of impact as a function of time has clinical implications, as children and adolescents with PDE histories may “grow into” or “out of” impairment throughout development. Additional research with a larger sample size and a comprehensive measure of the social environment beyond ECE could help inform the timing and targets of interventions for children with PDE histories.

Second, counter to hypotheses and prior literature suggesting that female sex is associated with better cognitive outcomes (Moe and Slinning, 2001; Bennett et al., 2002, 2007; Delaney-Black et al., 2004; Buss et al., 2007; Nair et al., 2008; Samplin et al., 2013; Traccis et al., 2020), the associations between hippocampal volume, ECE, and memory were moderated by sex at age 18 but not age 14. Little is known about how the effects of PDE may vary by biological sex throughout development (Conradt et al., 2018). Contrary to our findings, some have suggested that sex differences in outcomes may decline with age and are more prominent earlier in development (Traccis et al., 2020). Literature has linked estrogen to the promotion of hippocampal neurogenesis and synapse formation, suggesting a potentially protective role of the hormone on hippocampal development (Cooke and Woolley, 2005; Satterthwaite et al., 2014; Damme et al., 2020). To our knowledge, the association between estrogen and hippocampal development in samples of children with PDE has yet to be studied; however, given that the current study spans the pubertal period, fluctuations in estrogen may account for some sex differences. Overall, our findings suggest an enduring interaction between PDE biological sex, necessitating further research on sex-dependent outcomes.

Lastly, although we did see an association between memory performance at age 14 and hippocampal volume at age 18, we did not replicate previous findings that memory performance was associated with left hippocampal volume at age 14 (Riggins et al., 2012). One possible explanation may be using a comprehensive measure of memory, including verbal and nonverbal memory tasks, in the current study. It is also possible that potential variations in hippocampal volume were obscured because of the choice to estimate whole hippocampal volumes, rather than hippocampal subregions (Canada et al., 2022). An alternative explanation may be that current measures lack the specificity to show mechanistic differences in retrieval. For example, an fMRI study in this sample found differences between children with PDE and controls in memory encoding but not retrieval or performance (Geng et al., 2018). This finding suggests that overall performance may not differ between groups, but the neural resources necessary to support memory differ as a function of exposure. A better understanding of the consequences of exposure-dependent differences in neural resources and the relative impact of PDE on different stages in the memory process (e.g., encoding and retrieval) could inform future intervention targets.

### Strengths and limitations

The current study adds to the limited literature exploring the long-term impacts of PDE on outcomes in memory and its associated neural structure. It adds a critical piece – the impact of the early social world (early caregiving emotional environment). Moreover, the use of neural assessment at multiple time points and the investigation into potential sex differences provides further insight into why past findings may be mixed, suggesting that the effects of PDE may be age and sex-dependent.

Although our study significantly contributes to the literature in support of the combined risk model, suggesting that teratogenic and maternal risk factors interact to influence child behavioral outcomes (Konijnenberg et al., 2015; Schuetze et al., 2021), it also has methodological limitations that necessitate caution in the interpretation of results. The sample size was small, particularly at age 18, limiting statistical power; however, the sample size at age 14 is consistent with prospective longitudinal, neuroimaging studies of high-risk children, and attrition analyses demonstrated few differences in demographic characteristics, reducing the likelihood of bias. Although the sample is homogeneous, which limits our ability to generalize findings beyond this group, this homogeneity increases our ability to control for the confounds of racial discrimination, socioeconomic resources, and neonatal problems.

The present study is further limited by assessing outcomes only for children with PDE. A community comparison group was recruited around the six-year time point; however, comparison participants were not included in analyses due to the lack of measurement for the early caregiving environment. Another limitation of the present study is the high incidence of polysubstance use. 87% of participants were exposed to three or more substances, including opioids and stimulants. Although polysubstance use complicates our ability to attribute findings to a specific drug, 85% of longitudinal studies of PDE consist of polysubstance-exposed children, which is consistent with typical substance use behavior (Lester et al., 1998; Ackerman et al., 2010; Jaekel et al., 2021). Therefore, while it is impossible to separate out specific substances in this sample, findings from this study have high ecological validity and can be generalized to many other studies of children with PDE.

Additionally, it was outside of the scope of the present paper to evaluate the influence of adolescent peer relationships on outcomes. However, it is worth noting that the adolescent period is a time where peers become increasingly influential in development (Telzer et al., 2018). Literature has demonstrated the importance of peers influence on adolescent risk-taking behavior (Albert and Steinberg, 2011); however, to our knowledge, studies have not explored the impact of peers on memory development. Future studies should evaluate the relative impact of peer and caregiver socialization on longitudinal memory development.

This study’s construct validity benefits from using latent variable structures. ECE comprises parent-report measures using multiple gold-standard assessments, which increases the likelihood that the overall factor score represents a valid assessment of the environment. Moreover, the use of multiple, standardized measures of memory, including verbal and nonverbal indices, furthers past research that has focused mainly on individual subtests of larger cognitive assessments, single measures, or one type of recall (i.e., verbal) (Guo et al., 1994; Sundelin Wahlsten and Sarman, 2013; Konijnenberg et al., 2016; Konijnenberg and Melinder, 2022). To our knowledge, the latent variable created for this study is the most comprehensive measure of episodic memory among the existent eight studies of PDE and memory outcomes, affording more confidence in statistical conclusions regarding the construct.

Lastly, the present study does not include a measure of continued maternal substance use or ECE as participants age, making it difficult to determine the impact of the concurrent caregiving environment on child outcomes beyond infancy; however, exploratory analyses revealed that the effects of ECE were still significant after controlling for the number of caregiver changes between birth and age 7, suggesting that the early caregiving environment continues to be important, even after controlling for instability. Future research should evaluate the relative impact of early-life experiences and later-life experiences on memory ability and hippocampal volume.

Notably, the measures of ECE used in this study range from negative to neutral. While a more negative caregiving emotional environment may pose a risk to child development, it is important to acknowledge that many potential factors can support resilience. Interventions for at-risk children have shown efficacy in improving caregiver-child attachment relationships and parental sensitivity and positively influencing child behavior and biology (Bick and Dozier, 2013; Bernard et al., 2017; Roben et al., 2017). Overall, ECE represents a modifiable construct that may, in turn, influence the developmental consequences of PDE. Future research should explore sources of resilience and more positive indicators of ECE.

## Conclusion

Results from the present study highlight the interaction between prenatal and postnatal environments, suggesting a negative emotional caregiving environment may accentuate the effects of PDE on neurocognitive development, and these effects may be moderated by biological sex. This study contributes to the limited literature on the impact of pre- and postnatal factors on memory development in this population. Further, it extends past findings by including a neuroimaging assessment at 18 years of age. Although the sample size is small, we demonstrate that the effects of the interaction between pre- and postnatal factors vary from early-mid adolescence to late adolescence. Our finding that these effects are sex-dependent in late adolescence, but not mid-adolescence adds to the limited literature examining the interaction between PDE and sex through adolescence and into early adulthood. Findings can potentially influence intervention efforts for parent–child dyads coping with PDE significantly. The result sheds light on potential modifiable mechanisms of intergenerational transmission of risk. By supporting maternal functioning and the early caregiving environment, we may buffer against neurocognitive developmental risks associated with PDE.

## Data availability statement

The original contributions presented in the study are included in the article/Supplementary material, further inquiries can be directed to the corresponding author.

## Ethics statement

The studies involving humans were approved by Institutional Review Boards at the University of Maryland Baltimore and the National Institute on Drug Abuse Intramural Research Program. The studies were conducted in accordance with the local legislation and institutional requirements. Written informed consent for participation in this study was provided by the participants’ legal guardians/next of kin.

## Author contributions

BHK, TR, and MAC contributed to the conception and design of the study. BHK performed statistical analyses with contributions from ZC. BHK wrote the first draft of the manuscript. All authors contributed to the manuscript revision, read, and approved the submitted version.

## Footnotes

^1^While the constructs of positive and negative parenting are commonly used in the literature, it is important to acknowledge that these concepts are influenced by environmental demands and societal constructs. Therefore, negative parenting may be adaptive or protective in some contexts (Baumrind, 1972; Rious et al., 2019).
