# Supplementary material for: Early emotional caregiving environment and associations with memory performance and hippocampal volume in adolescents with prenatal drug exposure
Source: Front Behav Neurosci. 2023 Nov 20;17:1238172. doi: 10.3389/fnbeh.2023.1238172 (PMC10699310; doi:10.3389/fnbeh.2023.1238172)
Supplement: Supplementary file 1 [file Table_1.DOCX]

| **Author** | **Age in Years *M(SD*), Range^*^** | **Type of Prenatal Exposure** | **Sample Size PDE group (N)** | **Sample Size Comparison Group (CG) (N)** | **Memory Assessment** | **Results**** |
| --- | --- | --- | --- | --- | --- | --- |
| **Early Childhood** | | | | | | |
| Konijnenberg et al., 2016 | 4.35 | Methadone or Buprenorphine | 35 | 32 | NEPSY Narrative Memory Subscale | PDE<CG |
| Sundelin Wahlsten & Sarman, 2013 | 5.27 (.65), 5-6 | Buprenorphine | 25 | NA | McCarthy Scales of Children's Abilities | PDE< Standardized Norms |
| **Late Childhood** | | | | | | |
| Guo et al., 1994 | 9.90 (1.50), 7-12 | Opioids | 16 | 27 | Sternberg Memory Task | PDE<CG |
| Konijnenberg & Melinder, 2022 | 10.9 (0.6), 9-11 | Opioids | 20 | 21 | Test of Memory & Learning (TOMAL-2) | PDE<CG |
| **Early Adolescence** | | | | | | |
| Hurt et al., 2009 | 12.3 (1.3) | Cocaine | 55 | 65 | Incidental Word & Face Memory task | PDE=CG |
| Betancourt et al., 2011 | 12.3 (1.3) | Cocaine | 55 | 65 | Incidental Word & Face Memory task | PDE=CG*** |
| Riggins et al., 2012 | 14.26 (1.13) | Cocaine and/or heroin | 76 | 62 | Children’s Memory Scale (CMS) & California Verbal Learning Test–Child Version (CVCL-C) | PDE<CG |
| Betancourt et al., 2011 | 14.7 (0.9) | Cocaine | 55 | 65 | Incidental Word & Face Memory task | PDE=CG |
| **Late Adolescence** | | | | | | |
| Geng et al., 2018 | 17.11 (1.13) | Cocaine and/or heroin | 19 | 22 | Source Memory Paradigm | PDE=CG |
| Betancourt et al., 2011 | 17.5 (0.9) | Cocaine | 55 | 65 | Incidental Word & Face Memory task | PDE=CG^+^ |

^* Not all studies reported the age range of their sample. Age range is reported when given.^

^**PDE<CC indicates exposed participants performed worse than non-exposed participants on memory assessment. PDE=CC indicates no observed differences in memory performance between exposed and non-exposed groups.^

^***Betancourt et al., 2011 found a marginally significant effect of PDE on incidental face memory and a significant PDE by assessment number on incidental word memory, indicated that scores increased at a slower rate in the PDE group, compared to the control group.^

^+ Indicates marginal significance.^

**^Table 1.^** ^Summary of behavioral findings of the impact of PDE on memory performance from early childhood to late adolescence.^

**References**

Betancourt, L. M., Yang, W., Brodsky, N. L., Gallagher, P. R., Malmud, E., Giannetta, J. M., Farah, M. J., & Hurt, H. (2011). Adolescents with and without gestational cocaine exposure: Longitudinal analysis of inhibitory control, memory and receptive language. *Neurotoxicology and Teratology*, *33*(1), 36–46. https://doi.org/10.1016/j.ntt.2010.08.004

Geng, F., Salmeron, B. J., Ross, T. J., Black, M. M., & Riggins, T. (2018). Long-term effects of prenatal drug exposure on the neural correlates of memory at encoding and retrieval. *Neurotoxicology and Teratology*, *65*, 70–77. https://doi.org/10.1016/j.ntt.2017.10.008

Guo, X., Spencer, J. W., Suess, P. E., Hickey, J. E., Better, W. E., & Herning, R. I. (1994). Cognitive brain potential alterations in boys exposed to opiates: In utero and lifestyle comparisons. *Addictive Behaviors*, *19*(4), 429–441. https://doi.org/10.1016/0306-4603(94)90065-5

Hurt, H., Betancourt, L. M., Malmud, E. K., Shera, D. M., Giannetta, J. M., Brodsky, N. L., & Farah, M. J. (2009). Children with and without gestational cocaine exposure: A neurocognitive systems analysis. *Neurotoxicology and Teratology*, *31*(6), 334–341. https://doi.org/10.1016/j.ntt.2009.08.002

Konijnenberg, C., & Melinder, A. (2022). Verbal and nonverbal memory in school-aged children born to opioid-dependent mothers. *Early Human Development*, *171*, 105614. https://doi.org/10.1016/j.earlhumdev.2022.105614

Konijnenberg, C., Sarfi, M., & Melinder, A. (2016). Mother-child interaction and cognitive development in children prenatally exposed to methadone or buprenorphine. *Early Human Development*, *101*, 91–97. https://doi.org/10.1016/j.earlhumdev.2016.08.013

Riggins, T., Cacic, K., Buckingham-Howes, S., Scaletti, L. A., Jo Salmeron, B., & Black, M. M. (2012). Memory ability and hippocampal volume in adolescents with prenatal drug exposure. *Neurotoxicology and Teratology*, *34*(4), 434–441. https://doi.org/10.1016/j.ntt.2012.05.054

Sundelin Wahlsten, V., & Sarman, I. (2013). Neurobehavioural development of preschool-age children born to addicted mothers given opiate maintenance treatment with buprenorphine during pregnancy. *Acta Paediatrica*, *102*(5), 544–549. https://doi.org/10.1111/apa.12210
